# Sociodemographic, socioeconomic, clinical and behavioural predictors of body mass index vary by sex in rural South African adults-findings from the AWI-Gen study

**DOI:** 10.1080/16549716.2018.1549436

**Published:** 2018-11-30

**Authors:** Ryan G. Wagner, Nigel J. Crowther, F. Xavier Gómez-Olivé, Chodziwadziwa Kabudula, Kathleen Kahn, Memory Mhembere, Zola Myakayaka, Stephen Tollman, Alisha N. Wade, as members of AWI-Gen and the H3Africa Consortium

**Affiliations:** a MRC/Wits Rural Public Health and Health Transitions Research Unit, School of Public Health, Faculty of Health Sciences, University of the Witwatersrand, Johannesburg, South Africa; b Umeå Centre for Global Health Research, Division of Epidemiology and Global Health, Department of Public Health and Clinical Medicine, Umeå University, Umeå, Sweden; c Department of Chemical Pathology, National Health Laboratory Service and Faculty of Health Sciences, University of the Witwatersrand, Johannesburg, South Africa; d INDEPTH Network, Accra, Ghana

**Keywords:** Obesity, gender, South Africa, BMI, rural

## Abstract

**Background**: Despite increasing obesity in South African adults, data on the prevalence and determinants of body mass index (BMI) from rural communities, home to a significant proportion of the population, are scarce.

**Objectives**: To investigate overall and sex-specific determinants of BMI in a rural adult South African population undergoing rapid social and epidemiological transitions.

**Methods**: Baseline cross-sectional demographic, socioeconomic, anthropometric, clinical and behavioural data were collected between 2015 and 2016 from 1388 individuals aged 40–60 years and resident in the Agincourt sub-district of Mpumalanga province, a setting typical of rural northeast South Africa. A Health and Socio-Demographic Surveillance System (HDSS) underpins the sub-district and contributes to the Africa Wits-INDEPTH partnership for Genomic Studies (AWI-Gen). Linear regression was used to investigate univariate associations between log-transformed BMI and individual variables and multiple linear regression was used to investigate independent predictors of BMI overall and in sex-stratified analyses.

**Results**: Median BMI was significantly higher in females (28.7 kg/m^2^[95% CI 24.2–33.2] vs 23.0 kg/m^2^[95% CI 20.3–26.8];p < 0.001) with male sex associated with 17% lower BMI. In sex-stratified multiple linear regression models, compared to those never married, BMI was 7% higher in currently married males and 6% in currently married females. Current smoking in men and former smoking in women were associated with reductions in BMI of 13% and 26% respectively, compared with non-smokers. Higher educational attainment in women and higher socioeconomic status in men were both associated with higher BMI, while being HIV-positive and alcohol consumption in women were associated lower BMI.

**Conclusions**: Female sex strongly predicts higher BMI in this rural African population. While some predictors of higher BMI differ by sex, married individuals in both sexes had a higher BMI, suggesting that, in addition to developing sex-specific interventions to combat overweight and obesity, targeting married couples may result in reduction in population BMI.

## Background

Sub-Saharan Africa is undergoing major demographic and epidemiological transitions marked by an ageing population, persisting communicable diseases such as HIV and tuberculosis and increasing non-communicable diseases (NCDs) such as hypertension and diabetes [1–3]. Over the past 25 years, the levels of overweight and obesity, measured respectively as a body mass index (BMI) ≥ 25 kg/m^2^ and 30 kg/m^2^, have increased by more than 330%, contributing to the growing burden of NCDs on the continent [4]. Elevated BMI is an important risk factor for a number of NCDs including cardiovascular diseases, type 2 diabetes (T2D), musculoskeletal conditions and certain cancers. In South Africa, it has been suggested that obesity is responsible for at least 78% of cases of T2D, 68% of cases of hypertension, 45% cases of ischemic strokes and 38% of cases of ischemic heart disease [5].

The prevalence of obesity on the African continent is heterogeneous with southern Africa, a region comprising Botswana, Lesotho, Namibia, South Africa, Swaziland and Namibia, having the highest levels [6]. South Africa has one of the highest levels on the continent, with 7.6% of males and 36.8% of females over the age of 15 years estimated to be obese (in this age group, 41.3% and 68.5% of males and females respectively, are considered to be overweight or obese) [7]. These figures vary significantly both by ethnic group and by sex, with the latter not seen in high-income countries [8].

While sex appears to be a strong biological risk factor for excess weight [7], it is likely to be the interplay between this and sociodemographic, socioeconomic, clinical and behavioural determinants, including diet, history of alcohol use and sedentary lifestyle, that ultimately results in increased BMI. These determinants may therefore represent modifiable risk factors for obesity in high prevalence areas such as South Africa. While previous studies in South Africa have explored the prevalence and determinants of elevated BMI in younger individuals and urban dwellers [9,10], there are relatively few data on BMI among adults in rural areas [11–13], where a significant proportion of the population resides. Health services in these areas are limited, highlighting the need to intervene in at-risk individuals before NCDs develop.

In this study, we aimed to identify the risk factors for increased BMI, a key determinant for NCDs, in a rural South African adult population in the midst of demographic and epidemiological transitions. Given existing evidence that suggests sex is a strong determinant of obesity, we investigated these risk factors both overall and in subgroups defined by sex. These factors may represent targets for context-specific interventions aimed at halting and ultimately reducing overweight and obesity.

## Methods

### Study site

This study forms part of the multi-site Africa Wits-INDEPTH partnership for Genomic Studies (AWI-Gen), which took place in 6 sites across 4 sub-Saharan African countries, including two sites in rural South Africa. The data presented in this paper were obtained from the Agincourt Health and Socio-Demographic Surveillance System (HDSS), one of the rural South African sites [14–16]. The Agincourt HDSS comprises 450 km^2^ and 31 research villages and is located 500 km northeast of Johannesburg in rural Mpumalanga. Since 1992, the Agincourt HDSS, managed by the MRC/Wits Rural Public Health and Health Transitions Research Unit, has annually enumerated the entire population to capture vital events, including births, migrations and deaths. In 2015, the population of the HDSS was 120,000 individuals residing in approximately 20,000 households.

### Participant selection

All individuals aged 40 years or older as of 1 July 2014 who were documented in the 2013 census as permanently residing in the study site, were eligible to participate in this study.

Individuals who had previously participated in earlier studies investigating the interaction between HIV and NCDs [17,18] were invited to participate and, to supplement this group, a random sample of individuals aged 40–60 years was drawn from the 2013 Agincourt HDSS database. The sample was stratified by sex to achieve equal numbers of males and females. Participants were a subset of those who had been enrolled in Health and Ageing in Africa: a Longitudinal Study of an INDEPTH Community in South Africa (HAALSI) [19]. Of the 2000 individuals selected for recruitment, 1465 agreed to participate. This paper is based on the 1388 (94.7%) individuals on whom complete data on all study variables were available. The 77 individuals excluded due to missing data did not differ significantly by age, sex or BMI.

### Study procedures

Study visits took place between March 2015 and May 2016. All selected participants were visited at home by trained fieldworkers and invited to participate in the study. Informed consent was sought and, when provided, study participants were invited to come to the Agincourt HDSS laboratory on a pre-specified date. At the laboratory, a fieldworker administered a computer-aided personal interview (CAPI) which solicited sociodemographic, socioeconomic, behavioural and clinical data. Participants also underwent a series of tests and measurements, including height and weight. These were measured by trained research staff using a Harpenden digital stadiometer (Holtain Ltd, Crymych, Wales) and a digital Genesis Growth Management Scale (Genesis Pharmaceuticals, Johannesburg, South Africa), respectively. All participants who took part in the study at the Agincourt HDSS laboratory were given a coffee mug as a token of appreciation at the conclusion of their visit.

### Variable derivation

#### Outcome variable

BMI was calculated by dividing the mass (in kilograms) of the individual by the square of their height (in metres).

#### Exposure variables

Sociodemographic, socioeconomic, clinical and behavioural variables were selected based on evidence from existing studies as well as factors such as carbohydrate intake that might be expected to influence BMI.

#### Sociodemographic variables

Age was calculated at the date of the study visit using the reported date of birth. Marital status was determined by self-report: individuals were asked to report whether they were currently married or cohabitating, previously married or cohabitating or had never married nor cohabitated, with those who had never married nor cohabitated used as the reference group in regression analyses. Educational attainment was also self-reported with participants indicating their highest completed level of education from a selection of no formal education, primary education and tertiary education. No formal education was used as the reference category in regression analyses.

#### Socioeconomic variables

Employment status was self-reported. Individuals who were self-employed, full-time or part-time employed by someone else or informally employed were classified as employed; unemployed individuals were used as the reference category in regression analyses. Socioeconomic status (SES) quintiles were derived from self-reported household assets using principal components analysis. This method, widely used in HDSSs, constructs a household wealth index based on the type of material of which the main dwelling is built, type of ablution facilities, water and energy sources and ownership of livestock and modern assets. This wealth index can then be used as a proxy for SES [20,21]. The lowest quintile was used as the reference group in analyses.

#### Clinical variables

Individuals were classified as HIV-positive, HIV-negative or of indeterminate HIV status. They were defined as being HIV-positive if they reported being previously diagnosed with HIV or tested positive at the time of enrolment in the associated HAALSI study (determined by Vironostika Uniform 11 [Biomeriuex, France] screening assay), HIV-negative if they reported previously having tested negative or tested negative at the time of enrolment in HAALSI and indeterminate if they were unaware of their status and declined a test at the time of enrolment; anti-retroviral therapy use was self-reported. HIV- negative participants were used as the reference group in analyses. Three blood pressure measurements, two minutes apart, were taken on each participant. The mean of the second and third measurements was calculated and hypertension was defined as a mean blood pressure reading ≥ 140/90 mmHg at the time of the interview or a previous history of hypertension.

#### Behavioural variables

The CAGE criteria [22] were used to classify alcohol intake. This questionnaire is used to screen for alcohol dependence and may therefore be a useful proxy to quantify alcohol intake. Participants were classified as having no alcohol consumption, current non-problematic consumption or current problematic consumption, with those reporting no consumption used as the reference group. Individuals were classified as never smokers if they reported never having smoked tobacco products, former smokers if they had previously smoked tobacco products and current smokers if they smoked tobacco products at all, regardless of the frequency. Those who reported never having smoked were used as the reference group. Data on food intake were collected, including questions on consumption of carbohydrates such as juice, sugary beverages and bread, all of which could theoretically affect BMI. To ensure consistency in the estimation of food intake, participants were shown sample cards illustrating different food and beverage quantities and asked to identify, to the nearest whole number, the quantity they regularly consumed. The definitions of servings of fruit and vegetable and the quantity of drink consumed can be found in Supplementary Table 1. Total moderate-vigorous physical activity (MVPA) was calculated by summing the self-reported moderate and physical activity involved in occupation-, travel- and leisure-related activities to determine the total minutes of MVPA per week.

**Table 1.** Sociodemographic, socioeconomic, clinical and characteristics of Agincourt adults.

	Overall (n = 1388)	Females (n = 846)	Males (n = 542)	p-value
Age (years)	51 (46–56)	51.5 (46–56)	51 (45–56)	0.779
**Marital status**				**< 0.001**
Never married or cohabitated (%)	124 (8.9)	55 (6.5)	69 (12.7)	
Currently married or cohabitating (%)	938 (67.6)	514 (60.8)	424 (78.2)	
Divorced/widowed (%)	326 (23.5)	277 (32.7)	49 (9.0)	
**Education status**				**< 0.001**
No formal education (%)	382 (27.5)	265 (31.3)	117 (21.6)	
Primary education (%)	544 (39.2)	327 (38.6)	217 (40.0)	
Secondary education (%)	379 (27.3)	210 (24.8)	169 (31.2)	
Tertiary education (%)	83 (6.0)	44 (5.2)	39 (7.2)	
Unemployed (%)	881 (63.5)	549 (64.9)	332 (61.2)	0.17
**SES quintile**				**< 0.001**
1st quintile (%)	212 (15.3)	101 (11.9)	111 (20.5)	
2nd quintile (%)	330 (23.8)	188 (22.2)	142 (26.2)	
3rd quintile (%)	175 (12.6)	109 (12.9)	66 (12.2)	
4th quintile (%)	326 (23.5)	203 (24.0)	123 (22.7)	
5th quintile (%)	345 (24.9)	245 (29.0)	100 (18.4)	
Body mass index (BMI) (kg/m^2^)	26.1 (22.1–31.3)	28.7 (24.2–33.2)	23.0 (20.3–26.8)	**< 0.001**
HIV seropositive (%)	485 (34.9)	301 (35.6)	184 (34.0)	0.788
BMI (HIV positive) (kg/m^2^)	24.4 (21.1–28.6)	26.3 (23.0–31.1)	22.2 (19.8–25.1)	
BMI (HIV negative) (kg/m^2^)	27.3 (25.4–34.0)	30.1 (25.4–34.0)	23.8 (20.7–27.8)	
Current anti-retroviral therapy use (%)	255 (18.4)	154 (18.2)	101 (18.6)	0.058
**Smoking status**				**< 0.001**
Never smoker (%)	1118 (80.6)	837 (98.9)	281 (51.8)	
Current smoker (%)	145 (10.4)	2 (0.2)	143 (26.4)	
Former smoker (%)	125 (9.0)	7 (0.8)	118 (21.8)	
**Alcohol consumption**				**< 0.001**
No history of consumption (%)	879 (63.3)	698 (82.5)	181 (33.4)	
Current non-problematic consumption (%)	247 (17.8)	49 (5.8)	198 (36.5)	
Current problematic consumption (%)	19 (1.4)	2 (0.2)	17 (3.1)	
Former consumption (%)	243 (17.5)	97 (11.5)	146 (26.9)	
Bread consumption (slices/week)	16 (8–28)	16 (9–28)	16 (6–28)	**0.034**
Vegetable consumption (servings/week)	4 (2–8)	5 (3–8)	4 (2–6)	**0.001**
Fruit consumption (servings/week)	3 (1–6)	3 (0–6)	3 (1–6)	0.383
Sugary beverage intake (drinks/week)	2 (1–3)	2 (1–3)	2 (1–2)	0.439
Juice intake (days/week)	1 (0–2)	1 (0–2)	1 (0–2)	**0.013**
MVPA (minutes/week)	630 (200–1410)	600 (200–1380)	720 (200–1440)	0.391
Sleep (hours/day)	8.9 (8.0–9.7)	8.9 (8.0–9.6)	9 (8–10)	0.408
Self-reported diabetes (%)	52 (3.8)	32 (3.8)	20 (3.7)	0.929
Hypertension (%)	737 (53.1)	493 (58.3)	244 (45.0)	**< 0.001**

Continuous variables are described as medians and interquartile ranges. Mann Whitney U test used to compare continuous variables; chi-square test used to compare categorical variables. Kruskal-Wallis test used to compare BMI between subgroups defined by HIV status. MVPA-moderate to vigorous physical activity, SES-socioeconomic status

Data were imported into the Research Electronic Data Capture system (RedCap) [23].

### Statistical analysis

Continuous variables were reported using medians and interquartile ranges (IQR) and categorical variables were reported using percentages. Mann Whitney U tests were used to compare continuous variables in subgroups defined by sex, while the chi-square test was used to compare categorical variables in these subgroups. The Kruskal-Wallis test was used to compare BMI between subgroups defined by HIV status. Given the significant sex differences in some variables, further analyses were stratified by sex.

Univariate associations between BMI and individual continuous and categorical variables were investigated using linear regression. Scatter plots were used to investigate extreme values which, in the case of independent variables, were replaced with the median value.

### Multiple linear regression models

We fitted a series of regression models in which the relationship between sex and BMI was adjusted sequentially for several individual sociodemographic, socioeconomic, clinical and behavioural factors, which differed between males and females, to investigate whether any of these attenuated the effect of sex. A sex-adjusted multiple regression model in the entire sample and models stratified by sex were then fitted to establish factors associated with BMI. Independent variables with a p-value < 0.2 in univariate analyses were included in the multiple regression models. Dietary factors were also included in multiple regression models, even if they did not attain this level of significance, given their theoretical importance. BMI was log-transformed prior to linear regression analyses to improve normality and regression coefficients were exponentiated prior to reporting. For categorical variables, the exponentiated coefficients indicated percentage change in BMI for the category of interest compared to the reference category while for continuous variables, the exponentiated coefficient indicated the change in BMI for a unit change in the explanatory variable. P- values < 0.05 were considered statistically significant. Analyses were performed using STATA version 14.2 (StataCorp, College Station, TX, USA).

### Ethical considerations

All participants in this study provided written, informed consent. This study was approved by the Human Research Ethics Committee (Medical) of the University of the Witwatersrand (clearance numbers: M121029; M170880) and the Research and Ethics Committee of the Mpumalanga Province Department of Health.

## Results

Approximately 61% of our sample was female with a median age of 51 years; over 60% were also unemployed (Table 1). There were significant differences between females and males in the distributions of marital status, education status, socioeconomic quintiles, smoking status and alcohol consumption and females were significantly heavier (p < 0.001). Consumption of bread, vegetables and juice also differed between sexes. More than one-third of the sample was HIV-positive, but the prevalence did not differ significantly between males and females. BMI was significantly lower overall in participants with HIV (p < 0.001) and in both females (p < 0.001) and males (p = 0.001) with HIV (Table 1). A significantly greater proportion of females had hypertension, while self-reported diabetes, another condition which may result from excess weight, had a low overall prevalence and did not differ between sexes.

In univariate analyses (Table 2), marital status, education status and socioeconomic status were associated with increases in BMI. The highest percentage increases in BMI were associated with being divorced or widowed (16%) and being in the highest socioeconomic quintile (15%). Despite statistically significant associations between higher BMI and consumption of bread, vegetables, fruit, sugary beverages and juice, the changes in BMI with unit increases in these dietary variables were negligible. Current smoking and current problematic alcohol consumption were associated with 14% and 15% reduction in BMI respectively, while HIV-positivity was associated with an 8% reduction in BMI and the use of anti-retroviral therapy was associated with a 12% reduction in BMI. In analyses stratified by sex, being currently married, having tertiary education and having higher socioeconomic status were associated with increased BMI in both sexes, while being HIV-positive, being a former smoker and current problematic alcohol intake were associated with lower BMI in both sexes.

**Table 2.** Linear regressions showing univariate associations between body mass index and sociodemographic, socioeconomic, clinical and behavioural characteristics in Agincourt adults.

	Overall (n = 1388)			Females (n = 846)			Males (n = 542)		
Variable	exp (B)	95% CI	p- value	exp (B)	95% CI	p- value	exp (B)	95% CI	p- value
Age	1.00	1.00–1.00	0.075	1.00	1.00–1.00	0.091	1.00	1.00–1.00	0.472
**Marital status**									
Never married or cohabitated	ref	–	–	ref	–	–	ref	–	–
Currently married or cohabitating	**1.12**	**1.07–1.17**	**< 0.001**	**1.09**	**1.03–1.16**	**0.005**	**1.11**	**1.05–1.17**	**< 0.001**
Divorced/widowed	**1.16**	**1.10–1.21**	**< 0.001**	**1.08**	**1.01–1.15**	**0.018**	1.02	0.94–1.09	0.659
**Education status**									
No formal education	ref	–	–	ref	–	–	ref	–	–
Primary education	1.00	0.97–1.03	0.856	**1.05**	**1.01–1.09**	**0.012**	0.96	0.92–1.00	0.072
Secondary education	1.00	0.97–1.03	0.907	1.04	1.00–1.08	0.084	1.00	0.95–1.05	0.939
Tertiary education	**1.07**	**1.01–1.13**	**0.023**	**1.10**	**1.03–1.18**	**0.007**	**1.08**	**1.01–1.17**	**0.034**
**Employment status**									
Unemployed	ref	–	–	ref	–	–	ref	–	–
Employed	1.02	0.99–1.04	0.215	1.01	0.97–1.04	0.743	**1.05**	**1.02–1.09**	**0.005**
**SES quintile**									
1st quintile	ref	–	–	ref	–	–	ref	–	–
2nd quintile	1.02	0.98–1.06	0.451	0.99	0.94–1.05	0.765	1.00	0.95–1.05	0.960
3rd quintile	**1.07**	**1.03–1.12**	**0.002**	1.04	0.98–1.10	0.254	1.05	0.99–1.12	0.090
4th quintile	**1.09**	**1.05–1.14**	**< 0.001**	**1.05**	**1.00–1.11**	**0.062**	**1.08**	**1.02–1.13**	**0.005**
5th quintile	**1.15**	**1.10–1.19**	**< 0.001**	**1.07**	**1.02–1.13**	**0.009**	**1.15**	**1.09–1.22**	**< 0.001**
HIV seropositive	**0.92**	**0.89–0.94**	**< 0.001**	**0.90**	**0.87–0.93**	**< 0.001**	**0.94**	**0.90–0.97**	**< 0.001**
Current anti-retroviral therapy use	**0.88**	**0.80–0.97**	**0.008**	0.95	0.86–1.05	0.301	**0.77**	**0.63–0.94**	**0.010**
**Smoking status**									
Never smoker	ref	–	–	ref	–	–	ref	–	–
Current smoker	**0.76**	**0.73–0.78**	**< 0.001**	1.14	0.84–1.55	0.402	**0.83**	**0.80–0.86**	**< 0.001**
Former smoker	**0.86**	**0.82–0.89**	**< 0.001**	**0.71**	**0.60–0.83**	**< 0.001**	**0.96**	**0.92–1.00**	**0.037**
**Alcohol consumption**									
No history of consumption	ref	–	–	ref	–	–	ref	–	–
Current non-problematic consumption	**0.83**	**0.80–0.86**	**< 0.001**	0.94	0.88–1.00	0.061	**0.90**	**0.86–0.93**	**< 0.001**
Current problematic consumption	**0.75**	**0.68–0.83**	**< 0.001**	**0.71**	**0.52–0.96**	**0.028**	**0.85**	**0.77–0.93**	**0.001**
Former consumption	**0.92**	**0.89–0.95**	**< 0.001**	0.97	0.93–1.02	0.222	0.97	0.93–1.02	0.236
Bread consumption	**1.00**	**1.00–1.00**	**< 0.001**	**1.00**	**1.00–1.00**	**0.014**	**1.00**	**1.00–1.00**	**< 0.001**
Vegetable consumption	**1.00**	**1.00–1.01**	**0.013**	1.00	1.00–1.01	0.111	1.00	1.00–1.00	0.927
Fruit consumption	**1.00**	**1.00–1.01**	**0.005**	1.00	1.00–1.01	0.062	**1.01**	**1.00–1.01**	**0.002**
Sugary beverage intake	**1.01**	**1.00–1.02**	**< 0.001**	**1.01**	**1.00–1.02**	**0.013**	**1.01**	**1.00–1.02**	**0.007**
Juice intake	**1.01**	**1.00–1.01**	**0.010**	1.00	1.00–1.01	0.268	1.00	1.00–1.01	0.307
MVPA	1.00	1.00–1.00	0.585	1.00	1.00–1.00	0.805	1.00	1.00–1.00	0.833
Sleep	**0.99**	**0.98–1.00**	**0.004**	1.00	0.99–1.01	0.685	**0.98**	**0.97–0.99**	**< 0.001**

Exp (B)- exponentiated regression coefficient; coefficient interpreted as percentage change in BMI for category of interest vs reference category for categorical variables and percentage change in BMI for unit change of independent variable for continuous variables; MVPA- Moderate to Vigorous Physical Activity; SES-socioeconomic status

There was a strong association between sex and BMI. Being male was associated with a 17% reduction in BMI and the strength of this relationship was not attenuated by adjustment for any of a series of sociodemographic, socioeconomic, clinical or behavioural characteristics (Table 3).

**Table 3.** Linear regressions showing association between sex and body mass index in Agincourt adults, adjusted for individual sociodemographic, socioeconomic, clinical and behavioural characteristics.

Variable	exp (B)	95% CI
Sex alone	0.83	0.81–0.85
Sex adjusted for marital status	0.83	0.81–0.85
Sex adjusted for education status	0.82	0.80–0.84
Sex adjusted for SES quintile	0.84	0.82–0.86
Sex adjusted for current antiretroviraltherapy use	0.83	0.81–0.85
Sex adjusted for smoking status	0.88	0.86–0.90
Sex adjusted for alcohol consumption	0.86	0.84–0.88
Sex adjusted for bread slices/week	0.83	0.81–0.85
Sex adjusted for vegetables servings/week	0.83	0.81–0.85
Sex adjusted for juice days/week	0.83	0.81–0.85

Exp (B)- exponentiated regression coefficient; coefficient interpreted as percentage change in BMI for category of interest vs reference category for categorical variables and percentage change in BMI for unit change of independent variable for continuous variables; SES-socioeconomic status

In multiple linear regression models, several sociodemographic, socioeconomic, clinical and behavioural factors independently predicted BMI, with differing patterns in females and males (Table 4). In an overall model adjusted for sex, BMI was 7% higher in those who were currently or previously married when compared to those who had never been married, while being in the 4^th^ and 5^th^ socioeconomic quintiles was associated with a 5% and 6% increase in BMI respectively. In contrast, being HIV-positive was associated with a 6% reduction in BMI when compared to HIV-negative individuals, while current smoking and former smoking were associated with 14% and 5% reductions in BMI respectively. While bread consumption was statistically associated with an increased BMI, consumption of a single additional piece of bread weekly did not result in any meaningful change in BMI.

**Table 4.** Multiple linear regression showing associations between body mass index and sociodemographic, socioeconomic, clinical and behavioural characteristics in Agincourt adults.

	Overall^a^			Females			Males		
Variable	exp (B)	95% CI	p-value	exp (B)	95% CI	p- value	exp (B)	95% CI	p- value
Age	1.00	1.00–1.00	0.161	1.00	1.00–1.01	0.078	1.00	1.00–1.00	0.743
**Marital status**									
Never married or cohabitated	ref	–	–	ref	–	–	ref	–	–
Currently married or cohabitating	**1.07**	**1.03–1.11**	**0.001**	**1.06**	**1.00–1.13**	**0.042**	**1.07**	**1.02–1.12**	**0.008**
Divorced/widowed	**1.07**	**1.02–1.12**	**0.003**	**1.08**	**1.01–1.15**	**0.018**	1.03	0.96–1.10	0.434
**Education status**									
No formal education	ref	–	–	ref	–	–	ref	–	–
Primary education	1.01	0.98–1.04	0.526	**1.04**	**1.00–1.08**	**0.027**	0.96	0.92–1.00	0.056
Secondary education	1.02	0.99–1.05	0.259	1.04	1.00–1.09	0.062	0.97	0.93–1.02	0.207
Tertiary education	1.04	0.98–1.09	0.164	**1.08**	**1.00–1.16**	**0.042**	0.98	0.91–1.05	0.579
**Employment status**									
Unemployed	ref	–	–	–	–	–	ref	–	–
Employed	1.01	0.98–1.03	0.625	–	–	–	1.02	0.98–1.05	0.277
**SES quintile**									
1st quintile	ref	–	–	ref	–	–	ref	–	–
2nd quintile	1.00	0.96–1.03	0.942	1.00	0.95–1.05	0.951	1.00	0.95–1.04	0.873
3rd quintile	1.03	0.99–1.08	0.109	1.03	0.98–1.10	0.255	1.03	0.97–1.09	0.281
4th quintile	**1.05**	**1.01–1.09**	**0.008**	1.04	0.99–1.10	0.131	**1.06**	**1.01–1.11**	**0.021**
5th quintile	**1.06**	**1.02–1.10**	**0.002**	1.03	0.98–1.09	0.228	**1.10**	**1.04–1.15**	**0.001**
HIV-positive	**0.94**	**0.91–0.97**	**< 0.001**	**0.91**	**0.88–0.95**	**< 0.001**	0.97	0.93–1.01	0.162
Current anti-retroviral therapy use	0.94	0.86–1.02	0.141	0.96	0.88–1.06	0.463	0.84	0.70–1.01	0.063
**Smoking status**									
Never smoker	ref	–	–	ref	–	–	ref	–	–
Current smoker	**0.86**	**0.83–0.90**	**< 0.001**	1.17	0.87–1.58	0.295	**0.87**	**0.83–0.91**	**< 0.001**
Former smoker	**0.95**	**0.91–0.99**	**0.017**	**0.74**	**0.63–0.87**	**< 0.001**	0.97	0.92–1.01	0.123
**Alcohol consumption**									
No history of consumption	ref	–	–	ref	–	–	ref	–	–
Current non-problematic consumption	0.97	0.94–1.01	0.143	0.98	0.92–1.04	0.511	0.97	0.93–1.02	0.238
Current problematic consumption	0.92	0.83–1.01	0.083	**0.70**	**0.52–0.94**	**0.019**	0.96	0.87–1.06	0.412
Former consumption	0.99	0.96–1.03	0.692	0.99	0.95–1.04	0.654	1.00	0.96–1.05	0.983
Bread consumption	**1.00**	**1.00–1.00**	**0.024**	1.00	1.00–1.00	0.189	1.00	1.00–1.00	0.109
Vegetable consumption	1.00	1.00–1.00	0.323	**1.00**	**1.00–1.00**	**0.049**	1.00	0.99–1.00	0.373
Fruit consumption	1.00	1.00–1.00	0.409	1.00	1.00–1.00	0.749	1.00	1.00–1.00	0.360
Sugary beverage intake	1.01	1.00–1.01	0.052	**1.01**	**1.00–1.02**	**0.039**	1.00	1.00–1.01	0.294
Juice intake	1.00	0.99–1.00	0.786	1.00	0.99–1.01	0.993	0.99	0.99–1.00	0.242
MVPA	1.00	1.00–1.00	0.591	1.00	1.00–1.00	0.606	1.00	1.00–1.00	0.976
Sleep	1.00	0.99–1.01	0.694	–	–	–	0.99	0.98–1.00	0.245

^a^ Adjusted for sex; Exp (B)- exponentiated regression coefficient; coefficient interpreted as percentage change in BMI for category of interest vs reference category for categorical variables and percentage change in BMI for unit change of independent variable for continuous variables; MVPA- Moderate to Vigorous Physical Activity; SES-socioeconomic status

In models stratified by sex, women who were currently or previously married had similar increases in BMI (6% and 8% respectively), compared to those who were never married, whereas in males, only being currently married was associated with an increased BMI (7%). Smoking status was also associated with BMI in both sexes. Females who were former smokers had a reduction in BMI of 26% compared to those who had never smoked (p < 0.001) while current male smokers had a reduction in BMI of 13% (p < 0.001).

The effects of other independent variables were confined to either females or males. Primary and tertiary education were associated with higher BMIs (4% and 8% respectively) in females while females with HIV had a 9% lower BMI, independent of antiretroviral drug use, and females with current problematic drinking had a 30% reduction in BMI. Sugary beverage and vegetable intake were statistically associated with a higher BMI in females, but the BMI change with unit increases in consumption of these was negligible. The effect of socioeconomic status was seen only in males, with those in the 4^th^ and 5^th^ quintiles having a higher BMI (6% and 10% respectively) than those in the 1^st^ quintile.

## Discussion

In this study, we highlight the strong association of female sex with higher BMI in South African adults in a rural area. We illustrate that sociodemographic, socioeconomic, clinical and behavioural factors associated with BMI differ in females and males, with being currently or formerly married and having a tertiary education predicting the greatest increase in BMI in females and being in the highest socioeconomic quintile predicting the greatest increase in men.

Our study is in keeping with other work that has reported higher BMI in females. Previous studies in this rural population have reported higher BMIs in females, with an age-adjusted prevalence of obesity of 26% in females aged 15 and older compared to 7% in similarly aged males [17] and a 24.6% higher prevalence of obesity in females in those over 50 years [18]. Similar differences were evident in rural Ghana where the prevalence of obesity in women was seven times that in men [24]. The sex disparity in obesity appears independent of urbanicity with females in rural and peri-urban Uganda being 4.3 times as likely to be obese as males [25] and the prevalence of obesity in females 20–75 years of age in urban Cameroon being 4 times that of their male counterparts [26]. This female preponderance of obesity may be due to several factors including female perceptions of ‘ideal’ body weight, differential effects of childhood undernutrition and adult socioeconomic status [27], although in our study, socioeconomic status did not attenuate the relationship between sex and BMI.

We identified two factors that were associated with BMI in both sexes, namely marital status and smoking status. Our study supports previous research across multiple countries that reported currently married individuals have a higher BMI than their unmarried counterparts [28–30], although the relationship between a higher BMI and being married appears less robust in females. The reasons for this are not entirely clear but may relate to higher household income and ability to afford food. Our finding of increased BMI in previously married females however contrasts with other work which reported either no association or decreased weight, particularly in widowed females [28,31]. The association of decreased weight with widowhood in females is independent of age and changes in eating patterns relating to solitude may be responsible [32]. Societies in rural South Africa, in contrast to the high-income countries in which these studies were conducted, may provide for more mechanisms of social support after the death of a partner, reducing the effects of widowhood on eating patterns. Additionally, we categorised divorced and widowed females together in our study due to sample size and these transitions in marital status may have differing effects on BMI.

We found tobacco use to be inversely related to BMI, with former female smokers and current male smokers having a lower weight than those who had never smoked. Our findings are consistent with a large study in the UK which found that current smokers were 17% less likely to be obese than non-smokers [33]. Former smokers were overall more likely to be obese, but those former smokers who had smoked fewer cigarettes were less likely to be obese suggesting, as the study authors note, that there may be subgroup differences that were not evident in overall associations.

There were predictors of BMI that were restricted to either females or males. Higher educational attainment independently predicted increased BMI in female participants and higher socioeconomic status independently predicted increased BMI in males. The effect of both of these predictors on BMI appears to depend on the wealth and education status in the broader society. Higher BMI is often seen in the wealthier and better-educated in low and middle-income countries as evidenced by studies in peri-urban and rural Uganda and urban Nigeria and national South African studies [25,34,35]. In contrast, in high income countries, obesity appears to be more prevalent in poorer, less-educated individuals [36,37]. This may be due to several factors including the ability of wealthier individuals to afford larger quantities of food and perceptions in some communities in emerging economies that obesity is a desirable characteristic and indicative of personal wealth [38,39].

Being HIV-positive was also associated with lower BMI in females. Furthermore, the median BMI in HIV-positive females, while in the range for a normal BMI, was significantly lower than the median BMI in HIV-negative females. This association was independent of antiretroviral therapy use, suggesting that even when treated, individuals with HIV do not attain the weight of the background population. Several factors may contribute, including inadequate treatment. Use of antiretroviral therapy was self-reported in this study and we did not obtain measures of treatment adequacy. Additionally, more frequent contact with the health care system due to chronic HIV care may provide opportunities for reinforcement of health care messages on the importance of maintaining a healthy weight [40]. Finally, different antiretroviral therapies may also have differing effects on weight [41]. Lower BMI with problematic alcohol consumption may be due to the chronic malnutrition that can occur with excess alcohol intake [42].

While the associations between some dietary factors and BMI were statistically significant, the change in BMI was negligible suggesting that several units of change in these explanatory variables would be necessary to see significant changes in BMI. This contrasts with a study in another rural/peri-urban community in the Eastern Cape of South Africa where frequent consumption of fast food and fruit and low intake of vegetables were associated with increased risk of obesity [11]. Several factors may explain the absence of strong associations between dietary factors and BMI in our study. We did not, for example, assess consumption of dairy, animal protein, fast food or oils and fats, all of which may be related to BMI. Consumption of bread, juice and sugary beverages was also relatively low, suggesting that there may be other carbohydrate sources that contribute more significantly to the diet in this population and may therefore have a greater influence on weight. The study in the Eastern Cape included adults between 21 and 70 years and dietary influences on obesity may differ over such a broad age range; additionally, the quantification of intake associated with the broad categories of dietary intake of ‘never’, ‘sometimes’ and ‘always’ used in that study were unclear, making direct comparison with our study difficult.

## Limitations

This study provides important data on factors associated with BMI in rural South Africa. It does, however, have several limitations. Firstly, our participants were recruited from an HDSS and had been participants in previous studies of NCDs. While these previous studies were observational, participants with suspected hypertension were referred to local clinics. As such, their previous knowledge of their elevated blood pressure or attendance at clinics may have resulted in behavioural modification, making these participants less representative of the general, rural South African population. This limitation was somewhat mitigated by including a new, random sample of participants aged 40 to 60 years. We also performed a complete case analysis, but age, sex and BMI did not differ between the 94.7% of our respondents included in the analysis and those excluded. Thirdly, many of the behavioural exposure variables were self-reported and may therefore have been prone to recall or reporting bias. Some of our exposure variables may also have been too insensitive to identify subgroup effects- for example, the number of female smokers in our sample was quite small which may have resulted in an inability to detect an association between BMI and current smoking in women. We also did not quantify smoking and so were unable to investigate a relationship between the degree of smoking and BMI. We also defined alcohol consumption broadly and did not consider in detail the types and quantities of alcohol consumed; given the different caloric content of alcoholic beverages, a more precise determination of alcohol intake may have revealed other associations between alcohol intake and BMI. We also confined our analysis to the association between BMI and selected sociodemographic, socioeconomic, clinical and behavioural factors. There are likely several other exposure variables in this population that both directly influence BMI and confound the association between BMI and other factors and these were not investigated in this study. Our dietary variables, for example, were very broadly defined. A more precise determination of macronutrient composition may have allowed us to explore relationships between diet and BMI in more detail. Additionally, as HIV testing took place 1 day to 8 months prior to the study visit, some participants may have sero-converted and become HIV-positive in the intervening period. Given the cross-sectional nature of this study, we are unable to draw causal inferences. Future waves of data collection are planned in this cohort of individuals, which will allow better understanding of the causal relationship between BMI and sociodemographic, socioeconomic, clinical and behavioural determinants in females and males.

Our study sample was not nationally representative and the results are therefore not likely to be generalisable to the rest of the South African population. It does however share characteristics with other communities in rural South Africa which are undergoing epidemiological transition, including prevailing high mortality from HIV/AIDS and emerging increasing mortality from non-communicable diseases [21,43] and risk factors for obesity in this study may well be similar in these populations.

## Conclusion

This study has confirmed previous work in rural African populations identifying sex as a major determinant of obesity and identifies associations between BMI and sociodemographic, socioeconomic, clinical and behavioural factors that vary by sex. Other factors, such as being married, were associated with higher BMI in both sexes. While future longitudinal studies will assist in confirming the associations found in this study, public health interventions targeted at altering perceptions around the desirability of obesity in this and similar rural African communities and those aimed at married couples may impact weight in these populations.

## Paper context

Effective interventions are urgently needed to halt the increase in obesity in rural African populations, particularly given the scarcity of health resources to manage the consequent chronic non-communicable diseases. We demonstrate that while some predictors of higher body mass index in middle-aged and older adults in rural South Africa vary by sex, being married is a risk factor in both sexes and married couples may provide a target for public health interventions to reduce obesity.
