# Supplementary material for: Sociodemographic, socioeconomic, clinical and behavioural predictors of body mass index vary by sex in rural South African adults-findings from the AWI-Gen study
Source: Glob Health Action. 2018 Nov 30;11(Suppl 2):1549436. doi: 10.1080/16549716.2018.1549436 (PMC6282437; doi:10.1080/16549716.2018.1549436)
Supplement: Supplemental Material [file ZGHA_A_1549436_SM5993.docx]

**Supplementary Table 1: Definition of fruit, vegetable and drink servings**

| **One serving of fruit is equal to:** | 1 medium apple, 1 medium banana, 2 slices of mango, 1 medium pear, 1 medium orange, 1 medium naartjie, 1 handful of grapes, 1 slice of watermelon, 1 medium guava, 1 slice of pawpaw, 1 medium peach, 1 slice of melon |
| --- | --- |
| **One serving of vegetable is equal to:** | 7 slices of beetroot, 2 heaped tbsp of spinach, 1 medium onion, 1 medium tomato, 8 florets of cauliflower, 1 dessert bowl lettuce, 5 cm cucumber, ½ a pepper, 14 button mushrooms, 2 spears broccoli, 4 heaped tbsp green beans, 1 corn cob, 1 large sweet potato, 3 heaped tbsp cooked pumpkin/butternut 1/3 of a brinjal/egg plant |
| **One alcohol drink is equal to:** | 1 beer can/bottle (330ml), 1 glass of wine (125ml), 1 tot of liquor (25ml), 1L of traditional beer, 1L/carton of ijuba^¶^ |
| **One soft drink/juice is equal to:** | 1 cup or 250ml |

tbsp = tablespoon; ml = milliliter; L = litre; ^¶^local fermented beverage
